# Successful Management of a Posterior Post-Infarction Ventricular Septal Defect and Mitral Regurgitation with Delayed Surgery—A Case Report and Overview of the Literature

**DOI:** 10.3390/reports8020087

**Published:** 2025-06-04

**Authors:** Mihai Ștefan, Mircea Robu, Cornelia Predoi, Răzvan Ilie Radu, Daniela Filipescu

**Affiliations:** 1 Department of Anaesthesiology and Intensive Care, “Prof Dr CC Iliescu” Emergency Institute for Cardiovascular Diseases, 022328 Bucharest, Romania; 2 Faculty of Medicine, “Carol Davila” University of Medicine and Pharmacy, 050474 Bucharest, Romania; 3 Department of Cardiovascular Surgery, “Prof Dr CC Iliescu” Emergency Institute for Cardiovascular Diseases, 022328 Bucharest, Romania; 4 Department of Cardiology, “Prof Dr CC Iliescu” Emergency Institute for Cardiovascular Diseases, 022328 Bucharest, Romania

**Keywords:** ventricular septal defect, cardiogenic shock, invasive monitoring

## Abstract

**Background and Clinical Significance:** Ventricular septal defect (VSD) is a rare but serious complication following myocardial infarction (MI) that can lead to cardiogenic shock and carries a high mortality rate. Acute mitral regurgitation (MR) is another severe complication of MI with additional risks of mortality. The optimal timing of surgical intervention for VSD with MR is still being debated, and delaying surgery in medically manageable patients has been associated with improved survival. However, managing these patients in the intensive care unit (ICU) presents unique challenges. **Case Presentation:** In this paper, we present the case of a 52-year-old male with comorbidities who developed post-MI VSD with severe MR and underwent successful delayed surgical repair and mitral valve replacement. Our aim is to highlight the clinical characteristics, diagnostic approach, and management strategies of this rare complication in the critical care setting. The patient presented in cardiogenic shock and acute pulmonary edema. After stabilization using an intra-aortic balloon pump, pre- and afterload reducing pharmacotherapy and non-invasive mechanical ventilation, a watchful waiting strategy was employed, and surgery was performed on day 21 after hospital admission. Surgery was performed under general anesthesia, and the patient did not develop any complications related to the intra-aortic balloon pump or novel organ dysfunction. **Conclusions:** This case highlights the importance of a multidisciplinary approach to managing post-MI VSD with MR and emphasizes the need for careful patient selection and timing of surgical intervention in the critical care setting. Clinicians should be aware of the potential benefits of delaying surgical intervention in medically manageable patients, while also considering the unique challenges of managing these patients in the ICU.

## 1. Introduction and Clinical Significance

Ventricular septal rupture (VSD) following myocardial infarction (MI) is a rare but serious complication that demands prompt recognition and urgent treatment [1]. VSD leads to cardiogenic shock, most often through acute right heart failure due to left-to-right shunting, followed by end-organ, irreversible dysfunctions, and carries a high mortality rate [2,3]. Also, acute mitral regurgitation (MR) due to involvement of the posterior papillary muscle is an additional severe complication of MI, with incremental risks for mortality. While VSD or papillary muscle rupture may occur independently after MI, the concurrence of both lesions, while described in case reports and series, has an unknown incidence [4,5]. There has been a long-lasting debate regarding the timing of surgical intervention. While early surgery has a very high mortality, patients in cardiogenic shock who cannot be medically managed or assisted via temporary mechanical circulatory support (MCS) have a formal indication for emergency surgery [6]. However, the literature suggests that in patients who can be medically managed, postponing surgery is associated with improving survival in this critically ill population [7,8]. There is, however, a special challenge in adequately managing these patients in the intensive care unit (ICU), avoiding aggravated acute heart failure and end-organ dysfunction, and thus maximizing chances of survival. In this manuscript, we present the case of a patient who developed post-MI VSD, with concomitant severe mitral regurgitation (MR), and underwent delayed successful surgical repair and mitral valve replacement after 21 days of circulatory support in the ICU. Our aim is to underscore the clinical characteristics, diagnostic approach, and management strategies of this uncommon but critical complication in the critical care setting.

## 2. Case Presentation

A 52-year-old male, with known type II diabetes, essential arterial hypertension, and peripheral artery disease (PAD), with a history of limb revascularization via right femoral-tibial bypass and prior distal amputation of the second toe, presented to the emergency room of a tertiary, academic hospital at approximately 5 days after an episode of severe chest pain, with aggravated dyspnea for several hours.

**Figure 1:**
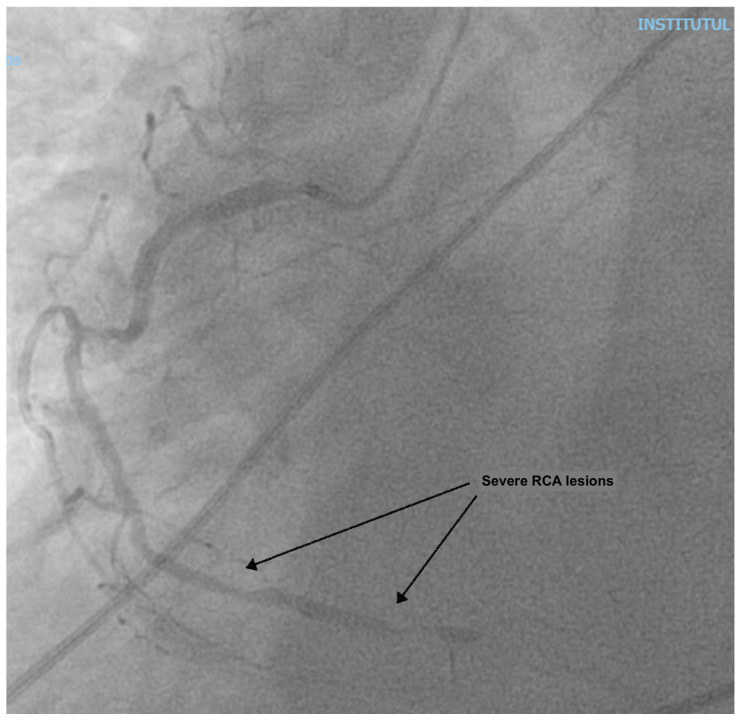
Coronary angiography showing an RCA sub-occlusion.

**Figure 2:**
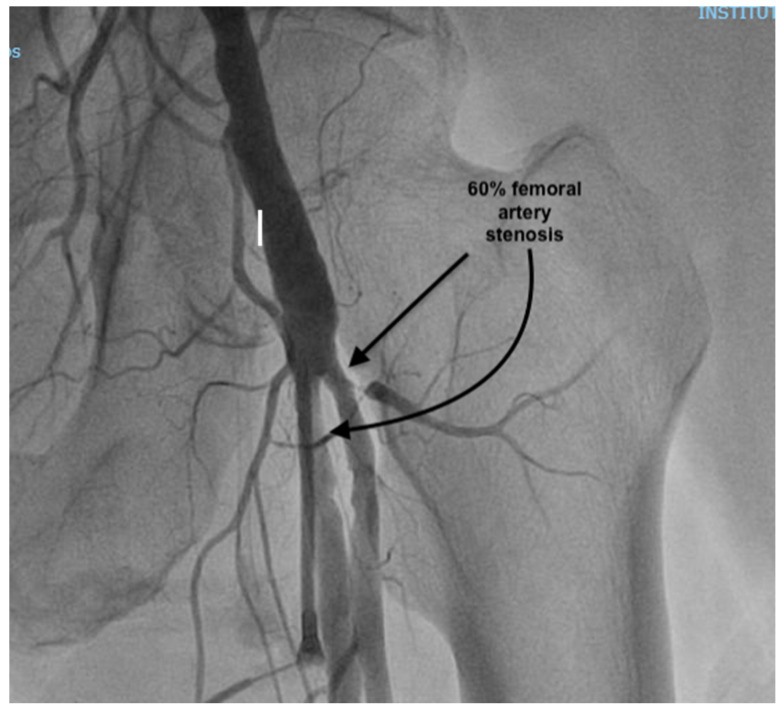
Peripheral angiography prior to IABP placement, showing 60% stenosis of superficial femoral artery and profonda femoris artery.

At presentation, the patient was in cardiogenic shock, at Society for Cardiovascular Angiography and Interventions (SCAI) stage C, with acute pulmonary edema: he was dyspneic, with a breathing rate of 25/min; an SpO_2_ of 90% under 6 L/min of oxygen, with diffuse fine crackles in both lungs; a blood pressure (BP) of 120/80 mmHg; a heart rate of (HR) 120/min; warm, but clammy skin; and a serum lactate of 2.5 mmol/L. He had a loud holosystolic murmur at cardiac auscultation. The ECG showed inferior ST-elevation myocardial infarction (STEMI), and the fast trans-thoracic echocardiography (TTE) showed moderate left ventricular (LV) dysfunction, with an ejection fraction (EF) of 40%, a moderate MR (vena contracta 6 mm), and a large VSD (2 cm), with left-to-right shunting. The patient was taken to the cardiac catheterization laboratory, where a right coronary artery (RCA) sub-occlusion was diagnosed (Figure 1), with no other hemodynamically significant coronary lesions. During the procedure, the patient was managed using non-invasive pressure-support mechanical ventilation (NIV) due to aggravated dyspnea and orthopnea. Interventional revascularization was not possible or indicated due to the distal lesions, with TIMI II flow, and the lack of benefit in an already non-viable myocardial territory. Despite significant PAD of the left inferior limb (Figure 2), an intra-aortic balloon pump (IABP) via the superficial left femoral artery was placed immediately.

Following the transfer to the cardiac intensive care unit (ICU), the patient was monitored invasively using a radial arterial line and a pulmonary artery catheter (PAC), which showed a pulmonary capillary wedge pressure (PCWP) of 23 mmHg, with a pulmonary artery pressure of 45/25 and a central venous pressure (CVP) of 17 mmHg, with high measured cardiac output via thermodilution in the context of a large left-to-right shunt. Repeated TTEs showed signs of right ventricle (RV) overload, and the patient was therefore decongested with intravenous (iv) loop diuretics (10 mg/h furosemide) and low-dose nitrates (lowering preload), while maintaining a 1:1 IABP assistance for LV afterload reduction. After this treatment and continuous NIV, the resolution of cardiogenic shock and pulmonary edema occurred at 6 h after ICU admission.

**Figure 3:**
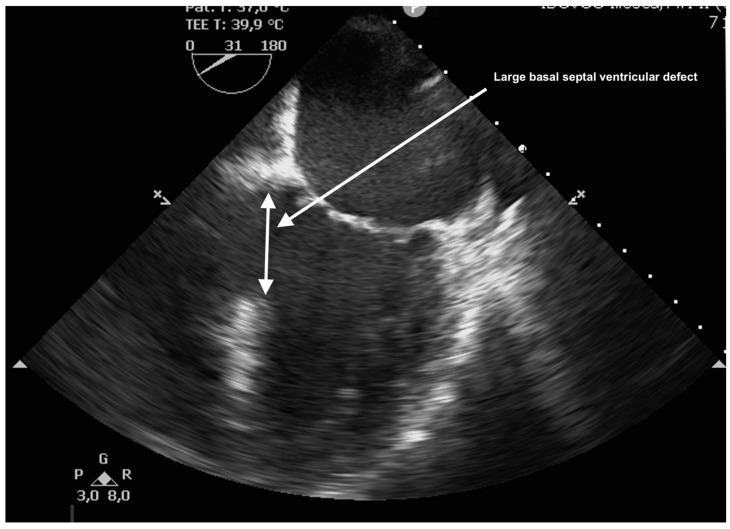
TEE showing large basal VSD.

**Figure 4:**
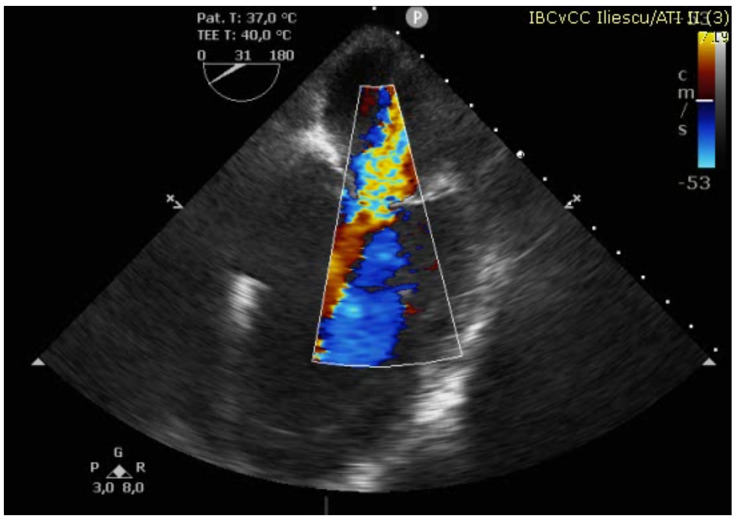
TEE showing severe MR and VSD.

The case was discussed in the multidisciplinary heart team (cardiologist, interventional cardiologist, cardiac surgeon, anesthesiologist) and, after informed consent, it was decided for a watchful waiting tactic, planning a delayed repair. Preoperatively, the patient was evaluated with a transesophageal echocardiography (TEE) for better defect characterization and MR quantification (Figure 3 and Figure 4). Percutaneous defect closure was deemed technically unfeasible due to the anatomy of the defect, which was located at the base of the septum and involved the mitral valve apparatus, while also exhibiting significant mitral valve regurgitation.

Surgery was performed on day 21 after hospital admission. The patient had not developed any complications related to the IABP or novel organ dysfunction.

**Figure 5:**
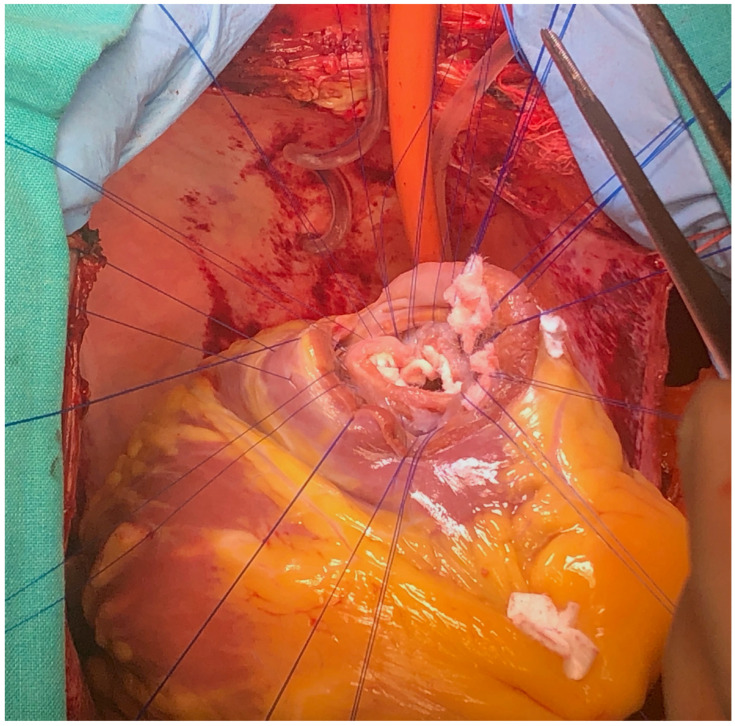
Surgical technique detail—exposure of VSD.

**Figure 6:**
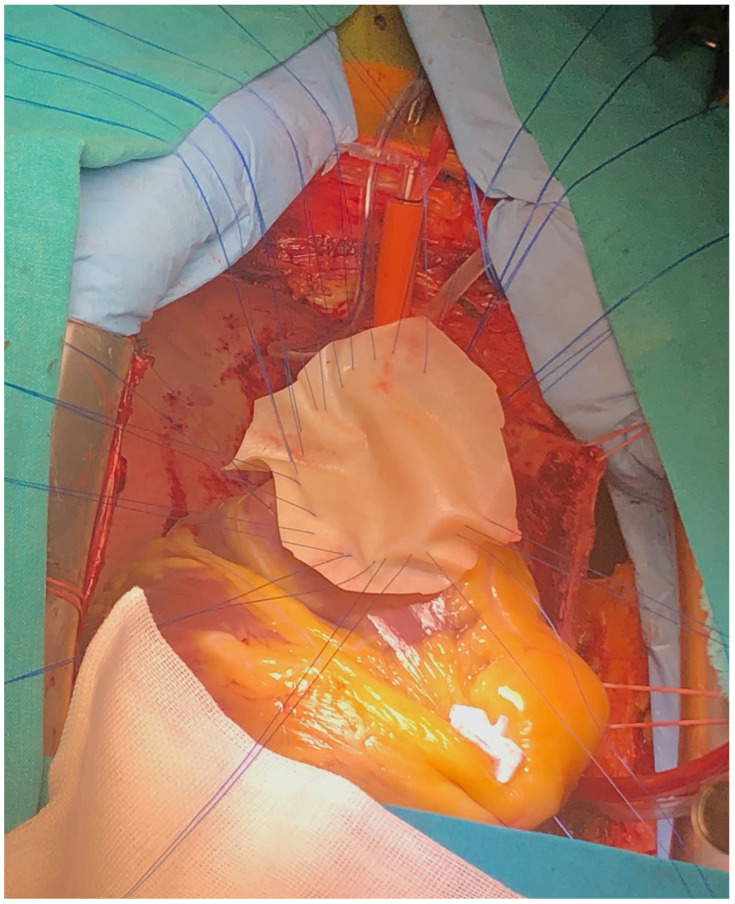
Surgical technique detail—large bovine pericardium patch.

**Figure 7:**
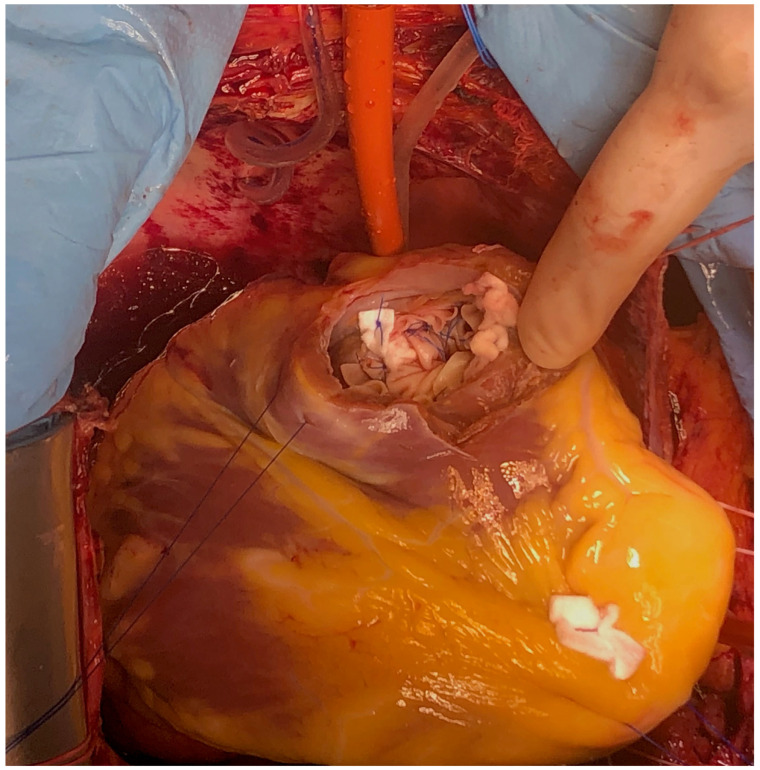
Surgical technique detail—VSD suture finished.

**Figure 8:**
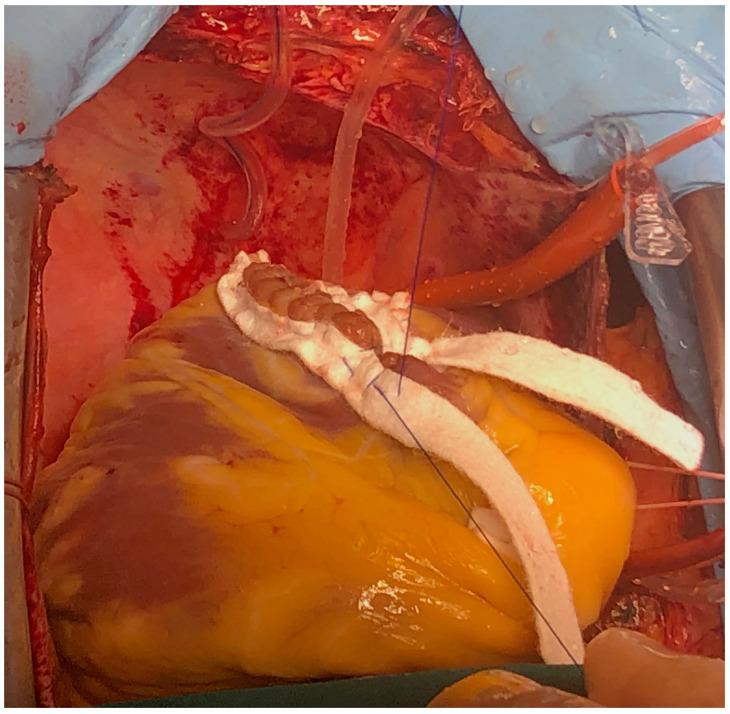
Intraoperative technique showing VSD repair with bovine pericardial patch (surgical technique described in text).

The surgery was carried out under general anesthesia, with iv induction and inhaled sevoflurane maintenance and total iv anesthesia during CPB. After median sternotomy, bicaval CPB was started, and the heart was arrested using anterograde and retrograde cold blood cardioplegia. After snaring both venae cava, the heart was enucleated with a 3.0 polypropylene stitch at the apex. A 10 cm incision was made on the posterior surface of the left ventricle, 2 mm parallel to the posterior interventricular artery. The margins of the VSD were identified. Interrupted matrass sutures of 2.0 Tevdek were placed circumferentially around the defect and used to secure a 4 × 7 cm bovine pericardial patch. The posterior papillary muscle was retracted into the sutures, so the mitral valve was replaced with a 27 biological prosthesis with preservation of the sub-valvular apparatus. The left ventriculotomy was closed with a double layer surjet suture, reinforced with Teflon felts. Details with surgical technique are shown in Figure 5, Figure 6, Figure 7 and Figure 8.

Other intraoperative strategies included the use of modified ultrafiltration, prophylactic tranexamic acid continuous infusion, a thromboelastometry-guided hemostasis management, and temporary inhaled nitric oxide administration during CPB weaning. The patient was transfused intraoperatively with two units of packed red blood cells and two units of fresh frozen plasma. Weaning was uneventful, under low-dose inotropes, and, postoperatively, the patient was transferred to the ICU.

After re-admission to the ICU, the patient had a good outcome under low-dose inotropes (dobutamine 5 mcg/kg/min and norepinephrine 0.1 mcg/kg/min), with a cardiac index of >3 L/min/m^2^ and a cardiac power output (CPO) of around 1 watt, while requiring temporary epicardial pacing for complete heart block. At echocardiography, he had a good myocardial contractility, a normal functioning mitral prosthesis, and no residual shunting. He was extubated after 11 h postoperatively and had an uneventful evolution, except for a hospital-acquired pneumonia and the persistent heart block, for which a permanent DDDR pacemaker was implanted on postoperative day 7. He was weaned off the IABP on postoperative day 4 and off inotropes on postoperative day 5. The patient was discharged to the ward on postoperative day 7.

He was discharged from the hospital after another 4 days in a good clinical condition.

## 3. Discussion

VSD is a rare but life-threatening complication of acute MI, with an incidence rate of 0.2–0.3% in the era of primary percutaneous coronary intervention (PCI) [1,6]. VSD and other mechanical complications of MI continue to be associated with high mortality rates, without reports of improvement in recent decades [9,10]. While medical management is not an option in these patients due to mortality rates approaching 100% [11], the optimal timing for surgical intervention in patients with VSD complicating AMI remains a topic of ongoing debate.

The European clinical practice guidelines remain ambiguous and do not give a firm recommendation as to the timing of surgery [12]. Performing surgery at an early stage is linked with a considerable mortality, which exceeds 50% in some cohorts [1,13], along with an increased chance of ventricular rupture reoccurrence [14]. On the other hand, delaying the surgery allows for easier septal repair in scarred tissue, but it poses the risk of end-organ dysfunction, rupture extension, and death while awaiting surgery [15]. Hence, it is recommended to conduct early surgery for patients with severe heart failure who do not respond promptly to aggressive medical treatment [16,17]. Delayed elective surgical repair can be an option for patients who respond well to heart failure therapy [18].

While delayed surgical intervention may improve the technical feasibility and allow myocardial tissue to fibrose, thereby reducing the risk of suturing through necrotic myocardium, it is not without significant risks. Prolonged reliance on intensive care and mechanical circulatory support increases the likelihood of nosocomial infections, ICU-acquired weakness, and device-related complications [19,20]. Furthermore, retrospective analyses from multicenter registries have shown mixed results regarding the timing of surgery. A study using the Society of Thoracic Surgeons (STS) database reported a mortality rate exceeding 50% for patients undergoing early VSD repair (<7 days post-MI), but this decreased to approximately 20–30% when surgery was delayed, provided that the patient could be stabilized [13]. However, this benefit must be weighed against the increased risk of preoperative deterioration, as up to one-third of patients awaiting delayed repair may die before reaching surgery [10].

In this case, the strategy was to stabilize the patient hemodynamically and medically while awaiting surgery. This approach can involve the use of various MCS devices, such as extracorporeal membrane oxygenation (ECMO), or Impella, which have been proposed as a bridge to surgery or percutaneous closure of the defect [21,22]. There are, however, no studies linking these newer devices with improved survival in mechanical complications after MI. Following the IABP-SHOCK II trial, in cases with MI presenting with cardiogenic shock, the use of IABP was downgraded to a IIIA in the ESC guidelines [14,23]. For mechanical complications, including VSDs, there is still a IIaC recommendation [12].

In the presented case, the patient was hemodynamically stabilized with the use of the IABP and optimized medical management before undergoing surgery on day 21 after hospital admission. This delayed strategy was chosen based on the high surgical risk (logistical Euroscore 52%) due to the patient’s comorbidities and the complexity of the VSD.

Given the inconclusive results from the literature, the timing of surgical intervention in VSD complicating AMI should be individualized, taking into account the patient’s clinical presentation, hemodynamic status, and comorbidities and the surgical center’s expertise. As for MCS, some suggest implantation of (veno-arterial) VA ECMO or Impella while preparing for surgery [21,24].

Mechanical circulatory support (MCS) plays a central role in stabilizing patients with post-infarction VSD and MR prior to surgical repair. However, the selection of an appropriate device must be tailored to the underlying pathophysiology, anatomical considerations, and institutional expertise. The intra-aortic balloon pump (IABP) remains the most widely used device in this context due to its relative ease of insertion, low cost, and ability to reduce afterload without increasing left-to-right shunting. Its use is particularly advantageous in patients with preserved systemic blood pressure and no severe aortic insufficiency [24]. In contrast, the Impella device, while effective in unloading the left ventricle, may exacerbate shunt flow across the VSD and is typically contraindicated in large septal defects due to ineffective circulatory support and the risk of paradoxical embolism [22].Veno-arterial extracorporeal membrane oxygenation (VA-ECMO) offers full biventricular support and oxygenation, making it attractive in cases of refractory cardiogenic shock or concomitant respiratory failure. However, it increases the left ventricular afterload and may worsen mitral regurgitation or shunting unless combined with additional LV decompression strategies. ECMO is also associated with significant risks, including bleeding, thromboembolism, and infection, and is more resource-intensive than IABP. Given these considerations, IABP may remain the first-line option in selected patients with post-MI VSD, while the use of Impella or ECMO should be restricted to centers with high MCS expertise and carefully selected indications. The development of cost-effective, tailored algorithms for MCS deployment in mechanical complications of MI remains an unmet need.

Furthermore, in this case, this would have been technically difficult. Also, given the lack of definitive trials in this context [25], the potential complications of devices such as ECMO have to be considered [19,20]. In this case, the increased afterload that is inherent to peripheral ECMO would have aggravated both the mitral regurgitation and the shunt fraction through the large defect, leading to unpredictable consequences. The IABP, coupled with careful medical management, proved to be optimal in the global approach of watchful waiting for the right surgical timing.

Early invasive monitoring can be useful in such cases for real-time hemodynamic interventions to optimize the pre- and afterload. In this case, while IABP was instituted at h1 after hospital admission, during the Cath-lab diagnostic procedure, early PAC monitoring allowed for targeted decongestion and avoidance of the vicious cycle of increased preload, RV dysfunction, cardiogenic shock, and end-organ dysfunction.

The utility of the PAC has been debated, its use in routine cardiac surgery has declined constantly, and there are some who would exclude it for good from clinical practice [26]. In our case, it proved invaluable for real-time monitoring of the response to fluid de-escalation and vasoactive manipulation of the preload and afterload, and this is supported by other authors [27]. Its use in experienced institutions and for selected patients, in conjunction with other, less invasive means of monitoring, can bring invaluable information for the management of complex patients [28,29].

While it is known that the cornerstone of treatment of AHF are diuretics and vasodilators [30], it is often difficult to initiate and further titrate these drugs in the case of patients presenting with cardiogenic shock, and even more so with mechanical complications of MI. While at hospital admission, our patient still had a preserved BP, we consider him to have been in shock, more precisely in class C of the Society for Cardiovascular Angiography and Interventions clinical expert consensus statement—hypoperfusion—which requires intervention, with lactate >2 mmol/L, extensive rales, clammy skin and a PCWP of more than 15 cmH_2_O [31]. This further underlines the role of managing such patients based on the pathophysiology and strict, real-time monitoring of the results of mechanical and/or pharmacological interventions.

Non-invasive ventilation was another tool used in this case for bridging this initially hypoxemic, tachypneic patient toward stabilization and delayed surgery. NIV is known to bring benefits in acute heart failure patients with pulmonary edema or cardiogenic shock by ameliorating the preload through increasing the intrathoracic pressure and reducing the venous return. It also modifies the mechanics of left ventricular afterload, by reducing the transmural pressure and systolic wall stress and lowering the end-diastolic LV pressure [32]. Additional benefits are derived from better aeration of fluid-filled alveoli, amelioration of ventilation–perfusion matching, and better oxygen uptake, and it was shown to improve vital signs and oxygenation and reduce the intubation rate in cardiogenic pulmonary edema complications following MI [33]. All these factors form a virtuous cycle in the increase of oxygen delivery to the heart and to the other tissues. In our case, the careful titration of PEEP and total airway pressure was required, as excessive pressures could lead to an acute increase in the RV afterload and additional strain on an already at-risk right heart.

Intraoperatively, the indication for mitral valve replacement was both due to functional and anatomical factors. Functionally, the patient had important left-heart side congestion and severe MR. Anatomically, due to the posterior papillary muscle being retracted into the sutures, even if there had not been preoperative MR, the surgical technique would have been identical.

During the ICU stay, the patient benefited from early mobilization, enteral feeding, and adequate psychological support, without developing end-organ dysfunction and allowing for clear lesion delimitation to provide optimal surgical technical conditions at the moment of the repair. As such, the postoperative ICU stay proved uneventful, except for the permanent bicameral pacemaker, which, given the anatomical particularity of the lesion, was not unexpected.

## 4. Conclusions

We present a case of successful delayed surgical repair of a post-infarction ventricular septal defect (VSD) with concomitant severe mitral regurgitation (MR), highlighting the potential benefits of a watchful waiting strategy in selected, medically manageable patients. Our report underscores the importance of early invasive monitoring, targeted pharmacologic stabilization, and a multidisciplinary heart team approach in optimizing the outcomes for this high-risk population. Future research should aim to better define the optimal timing of surgery by stratifying patients based on the defect size, anatomical location, hemodynamic status, and comorbidity burden. Prospective studies or multicenter registries are also needed to compare early versus delayed surgical outcomes and to assess the utility of percutaneous closure devices—particularly in anatomically favorable lesions—as either a bridge to surgery or a definitive treatment. Furthermore, standardized protocols for selecting mechanical circulatory support (MCS) strategies and incorporating cost-effectiveness analyses could greatly enhance decision-making in these complex cases.

## Data Availability

The original contributions presented in the study are included in the article, further inquiries can be directed to the corresponding author.
